# The Evaluation of Toxicity Induced by Psoraleae Fructus in Rats Using Untargeted Metabonomic Method Based on UPLC-Q-TOF/MS

**DOI:** 10.1155/2017/6207183

**Published:** 2017-11-27

**Authors:** Yanyan Xu, Yiwei Zhao, Jiabin Xie, Xue Sheng, Yubo Li, Yanjun Zhang

**Affiliations:** ^1^State Key Laboratory of Modern Chinese Medicine, Tianjin University of Traditional Chinese Medicine, No. 88, Yuquan Road, Nankai District, Tianjin 300193, China; ^2^School of Chinese Materia Medica, Tianjin University of Traditional Chinese Medicine, 312 Anshan West Road, Nankai District, Tianjin 300193, China

## Abstract

Psoraleae Fructus is the dry and mature fruit of leguminous plant* Psoralea corylifolia* L., with the activity of warming kidney and enhancing yang, warming spleen, and other effects. However, large doses can cause liver and kidney toxicity. Therefore, it is necessary to evaluate the toxicity of Psoraleae Fructus systematically. Although traditional biochemical indicators and pathological tests have been used to evaluate the safety of drug, these methods lack sensitivity and specificity, so a fast and sensitive analytical method is urgently needed. In this study, an ultraperformance liquid chromatography coupled with quadrupole time-of-flight mass spectrometry (UPLC-Q-TOF/MS) method was used to analyze the metabolic profiles of rat plasma. The changes of metabolites in plasma samples were detected by partial least squares-discriminant analysis (PLS-DA). Compared with the control group, after 7 days of administration, the pathological sections showed liver and kidney toxicity, and the metabolic trend was changed. Finally, 13 potential biomarkers related to the toxicity of Psoraleae Fructus were screened. The metabolic pathways involved were glycerol phospholipids metabolism, amino acid metabolism, energy metabolism, and so forth. The discovery of these biomarkers laid a foundation for better explaining the hepatotoxicity and nephrotoxicity of Psoraleae Fructus and provided a guarantee for its safety evaluation.

## 1. Introduction

Psoraleae Fructus is the dry and mature fruit of the leguminous plant* Psoralea corylifolia* L., with pungent flavor, slightly bitter, and attribution to kidney and spleen meridian [1]. It has antitumor [2], antioxidant [3], and other effects and has been clinically used as a tonic to treat osteoporosis [4], vitiligo [5], and so forth. Regardless of its therapeutic effects, high doses of Psoraleae Fructus have been reported to cause liver damage in clinic [6], and animal studies have shown that Psoraleae Fructus decoction following oral administration to rats caused hepatotoxicity and nephrotoxicity [7–9]. Thus concerns have risen about the safe medication of Psoraleae Fructus and the toxicity induced has attracted increasing attention. Traditional toxicity assessment method using biochemistry and histopathology lacks sensitivity and accuracy [8]. Therefore, it is urgent to establish a rapid and effective method to investigate toxicity induced by Psoraleae Fructus.

Metabonomics is a new research technique developed following genomics, transcriptome, and proteomics. It has become an important part of systematic biology [10]. It reflects the overall changes of the body through the metabolic changes of plasma, urine, and so on [11]. With the continuous progress of the discipline, untargeted metabonomics has been widely used in drug safety assessment and toxicity prediction and provided valuable information for drug-induced cardiotoxicity, hepatotoxicity, and nephrotoxicity [12–14].

In this study, toxicity induced by Psoraleae Fructus was assessed by using nontargeted metabonomic approach based on UPLC-Q-TOF/MS after intragastric administration of aqueous extract of Psoraleae Fructus to rats. Potential biomarkers of hepatotoxicity and nephrotoxicity of Psoraleae Fructus were screened by including multivariate statistical analysis. The sensitivity and specificity of biomarkers were evaluated with a receiver operating characteristic (ROC) curve. Finally, through metabolomic pathway analysis (MetPA), we screened the metabolic pathways regarding the toxicity of Psoraleae Fructus to explain the biological significance.

## 2. Materials and Methods

### 2.1. Reagents and Chemicals

Psoraleae Fructus was purchased from Beijing Heyanling Drug Herb Co, Ltd. (Beijing, China), and authenticated by the corresponding authors. HPLC-grade acetonitrile and formic acid were purchased from Oceanpak (Gothenburg, Sweden) and ROE (USA), respectively. Purified water was purchased from Wahaha Company (Hangzhou, China). Normal saline was provided by Qidu Pharmaceutical Co, Ltd. (Shandong, China).

### 2.2. Preparation of Psoraleae Fructus Aqueous Extract

50 g, 150 g, and 250 g crushed Psoraleae Fructus powders were weighted, respectively, and were extracted with 10 times the amount of purified water for the first time by reflux for 1 hour, followed by extraction with 8 times the amount of purified water for the second time by reflux for another 1 hour. After filtration, the filtrates were merged and concentrated to a concentration of 1.0 g/mL, 3.0 g/mL, and 5.0 g/mL (equivalent to the crude drug), respectively. The aqueous extracts were stored under 4°C before administration.

### 2.3. Animal Experiment

Twenty-four male Wistar rats weighing 190–210 g were provided by the Institute of Hygienic Environmental Medicine, Chinese Academy of Military Medical Sciences [SCXK (jing) 2012-0001], and were fed in the Animal Center of Tianjin Institute of Radiology (Tianjin, China). Rats were housed in SPF grade animal room at the ambient temperature of 23 ± 2°C and humidity 35 ± 5% with a 12 h dark-light cycle. After one week of adaptive feeding, the rats were randomly divided into four groups: normal control group (NS group), low dose of Psoraleae Fructus group (PLD group), medium dose of Psoraleae Fructus group (PMD group), and high dose of Psoraleae Fructus group (PHD group). Rats were orally administered with Psoraleae Fructus extract once daily at the dosage of 5 g/kg, 15 g/kg, and 25 g/kg for seven consecutive days for PLD group, PMD group, and PHD group, respectively. Rats for NS group were given normal saline. This experiment was approved by the Animal Ethics Committee of Tianjin University of Traditional Chinese Medicine. All experimental procedures were conducted in accordance with Chinese national legislation and local guidelines.

### 2.4. Sample Collection

Before the samples were collected, all animals were fasted for 12 hours but allowed to drink water to avoid the effects of food on the final outcome. After administration of 7 days (Day 8, morning), blood from both control group and different dosage groups was sampled via abdominal aorta, of which 5 mL was collected into heparinized test tube, and centrifuged at 3000 rpm for 15 min at 4°C to separate the supernatant, then the supernatant was centrifuged at 3500 rpm for 8 min at 4°C, and the supernatant obtained at the end served as a plasma sample. The plasma samples were collected and stored at −80°C for the metabonomic analysis; another 5 mL of the whole blood was placed in ordinary test tube, centrifuging in accordance with the above conditions to obtain the serum sample for biochemical detection. After blood sampling, all animals were sacrificed and the heart, liver, and kidney of each administration group were quickly removed and soaked in 10% formaldehyde solution for hematoxylin and eosin (H&E) staining.

### 2.5. Biochemical Detection and Histopathological Evaluation

The levels of serum creatine kinase (CK), creatine kinase-MB (CK-MB), alanine aminotransferase (ALT), aspartate aminotransferase (AST), creatinine (Cr), and blood urea nitrogen (BUN) were measured by automatic biochemical analyzer (Hitachi, Japan). Heart, liver, and kidney tissues were subjected to H&E staining, and paraffin sections were dewaxed by xylene, hydrated, and stained with hematoxylin for 10 minutes. After differentiation, eosin staining, dehydration, being cleaned in xylene, and neutral resin mounting, histopathological evaluation was performed under optical microscope by 200 times magnification. The pathological changes of rat tissues were observed.

### 2.6. Metabonomic Studies

#### 2.6.1. Sample Pretreatment

Plasma samples stored at −80°C were taken and melted at room temperature. To 100 *μ*L of plasma, 300 *μ*L of acetonitrile was added, vortexed to mix for 1 min, and followed by ice-water bath ultrasonication for 10 min. The mixture was centrifuged at 13000 rpm for 15 min, and the supernatant was injected for UPLC-Q-TOF/MS analysis. 10 *μ*L of plasma was obtained from different dosage groups and mixed as QC samples. The preparation of QC samples followed the same procedure with that of the plasma samples.

#### 2.6.2. Method Validation

Before plasma samples analysis, QC samples were analyzed for method validation. Twenty chromatographic peaks were selected randomly as common peaks from chromatograms of QC samples, and RSDs of peak area and retention time were calculated to test the system precision, method repeatability, and sample stability. One QC sample was injected six times to monitor the system precision of UPLC-Q-TOF/MS. Six QC samples were then parallel processed and injected to evaluate the method repeatability. In addition, a QC sample was analyzed after being stored at 4°C for 0, 2, 6, 12, and 24 h, respectively, to ensure that the plasma samples were stable.

#### 2.6.3. Chromatographic and Mass Spectrometric Conditions

The data acquisition was performed on an UPLC-Q-TOF/MS system (Waters, USA). An aliquot of 5 *μ*L of processed plasma sample was injected and separated with an ACQUITY UPLC HSS C_18_ column (2.1 mm × 100 mm, 1.7 *μ*m, Waters). The column temperature was 40°C and the flow rate was 0.3 mL/min. The mobile phase was water (A, containing 0.1% formic acid) and acetonitrile (B, containing 0.1% formic acid) using gradient elution. The elution conditions were as follows: 0 min–0.5 min, 99% A; 0.5 min–2 min, 99% A–50% A; 2 min–9 min, 50% A–1% A; 9 min-10 min, 1% A; 10 min–10.5 min, 1% A–99% A; 10.5 min–12 min, 99% A.

Mass spectrometry was operated in the positive ion mode using electrospray ionization (ESI) source. High-purity N_2_ was used as the auxiliary spray ionization and desolvation gas with temperature set at 350°C. The gas flow rate was 10 mL/min for drying gas, 600 L/h for desolvation gas, and 50 L/h for cone gas. The nebulizing gas pressure was 310 kPa and the capillary ionization voltage was 2.1 kV. The reference ion ([M + H]^+^ = 556.2771) was used to ensure accuracy in the spectral acquisition and the quadrupole scan range was* m/z 50–*1000 Da.

#### 2.6.4. Data Processing and Statistical Analysis

The original data obtained by UPLC-Q-TOF/MS analysis was collected by Masslynx (Waters, USA) software, and the ion selection, peak alignment, peak matching, and filtering were carried out by the data processing system. After normalization, data were exported to SIMCA-P+12.0 software (Umetrics AB, Umea, Sweden) for partial least squares discriminant analysis (PLS-DA). Metabolite with variable importance plot (VIP) > 1 was screened as a potential biomarker related to toxicity of Psoraleae Fructus. An independent sample *t*-test was then performed using SPSS 17.0 software to determine the statistical significance of metabolites. By comparison with authentic standards, MS/MS fragmentation, and HMDB (http://www.hmdb.ca/), the significantly varied biomarkers were identified. Then, the biomarkers were optimized using the receiver operating characteristic (ROC) curve by SPSS. Finally MetPA (http://metpa.metabolomics.ca./MetPA/faces/Home.jsp) was utilized to describe the involved metabolic pathways of the biomarkers.

## 3. Results and Discussion

### 3.1. Serum Biochemical Assessment and Histopathological Examination

**Figure 1:**
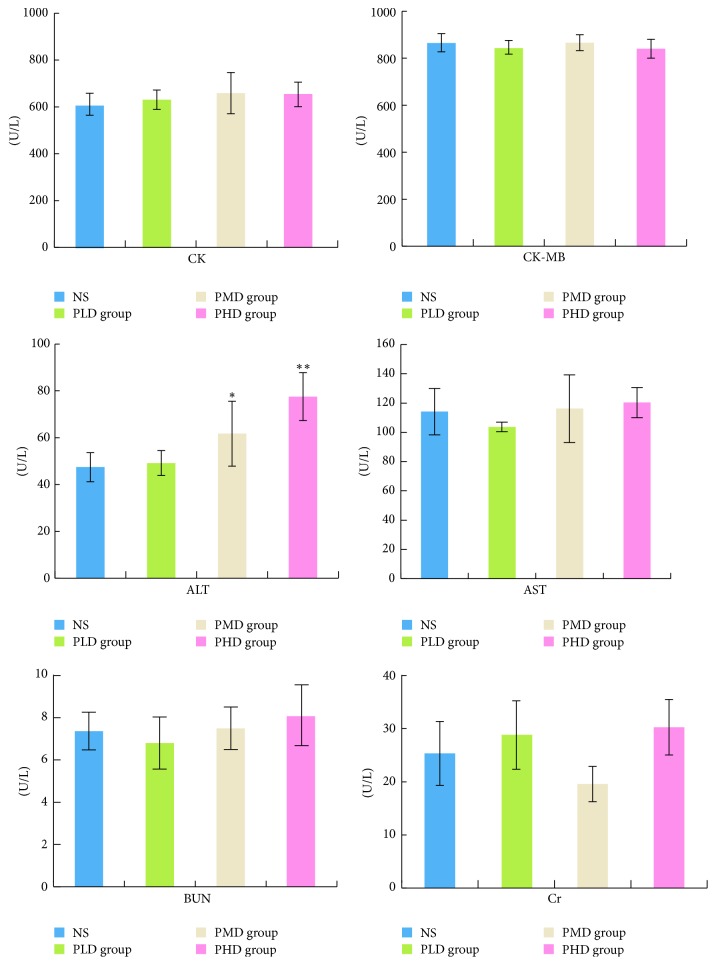
CK, CK-MB, ALT, AST, BUN, and Cr levels in serum samples (^*∗*^*P* < 0.05 and ^*∗∗*^*P* < 0.01, compared with the NS group).

Serum levels of CK, CK-MB, ALT, AST, BUN, and Cr in the administration group and NS group were compared, as shown in Figure 1. Compared to NS group, the levels of CK, CK-MB, AST, BUN, and Cr in the PLD, PMD, and PHD group were not significantly changed, while ALT concentrations of the PMD and PHD group were significantly increased, indicating liver injury.

**Figure 2:**
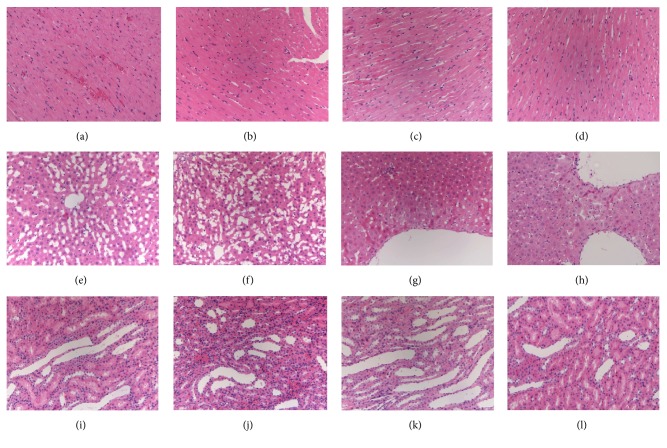
Histopathological examination of the heart, liver, and kidney by H&E staining (200x magnifications). (a) NS heart group, (b) PLD heart group, (c) PMD heart group, (d) PHD heart group; (e) NS liver group, (f) PLD liver group, (g) PMD liver group, (h) PHD liver group, (i) NS kidney group, (j) PLD kidney group, (k) PMD kidney group, and (l) PHD liver group.

Histopathological results are shown in Figure 2. No abnormality was observed for tissue sections of NS group. Compared with NS group, there was no obvious cardiac damage for PLD, PMD, and PHD group, as well as rare hepatic or renal damage for PLD group. But abnormal symptoms were observed for liver and kidney sections of PMD and PHD group. Hepatocellular cloudy swelling and degeneration and varying degrees of renal looser stroma and edema along with inflammatory cell infiltration and mild pyelectasis demonstrated that PMD and PHD group caused hepatic and renal injuries to rats.

### 3.2. Method Validation

System precision test showed that RSDs were lower than 12% and 1% for peak area and retention time, indicating good precision of the UPLC-Q-TOF/MS apparatus for metabolomic analysis. Method repeatability test showed RSDs within 14% and 1%, demonstrating precise sample preparation. Additionally, RSDs for peak area and retention time of plasma samples under storage conditions were less than 10% and 1%, revealing that processed samples were kept stable for 24 h during analysis. The results of method validation proved system precision, method repeatability, and sample stability all in line with the requirements of metabonomic studies.

### 3.3. Metabolic Profiling

**Figure 3:**
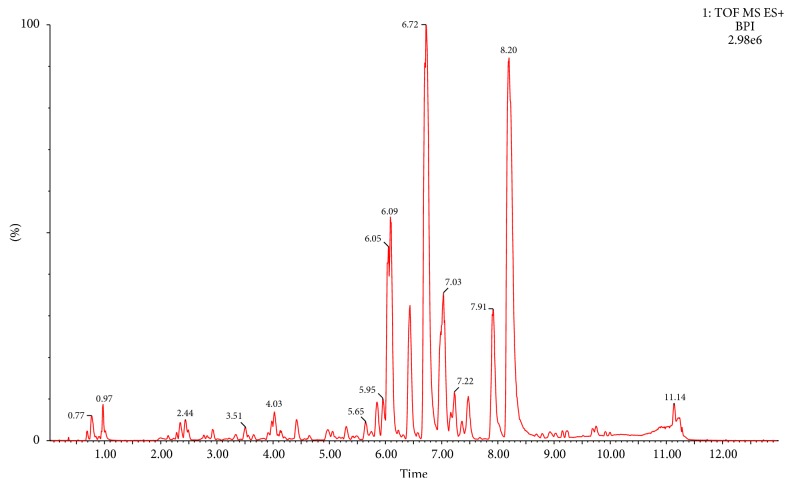
BPI chromatogram of a plasma QC sample.

UPLC-Q-TOF/MS technique was used to analyze the plasma samples of different dosage groups. The BPI of a plasma QC sample was shown in Figure 3.

**Figure 4:**
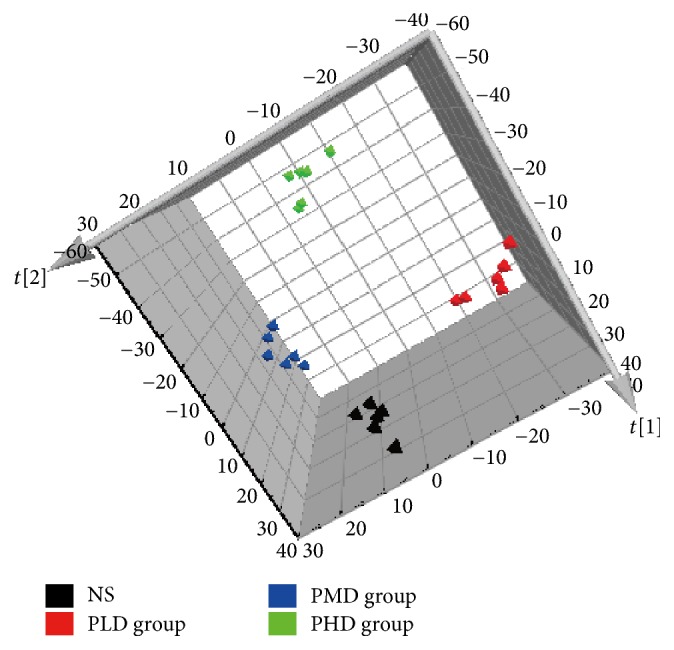
PLS-DA score plot for NS group and different dosage groups of Psoraleae Fructus extract.

The original data were analyzed by multivariate statistical analysis, using PLS-DA to determine the differential metabolites. It can be observed from the PLS-DA score plot (Figure 4) that administration groups were obviously separated from the control group, indicating that the drug administration caused changes of the endogenous metabolites. Generally speaking, in the PLS-DA model, the larger *R*^2^ and *Q*^2^, the more stable the model. For the PLS-DA model established in the present experiment, *R*^2^ and *Q*^2^ met the requirements, indicating that the established model was stable and reliable and could be used for further data analysis.

### 3.4. Identification of Biomarkers

Serum biochemical assessment and histopathological examination showed hepatic and renal injuries induced by PMD and PHD group in rats, but no abnormality was observed for PLD group. In order to find out potential biomarkers related to hepatotoxicity and nephrotoxicity, PLS-DA was performed. Based on the PLS-DA model, the small molecular metabolites with significant changes were screened, and the variables of VIP > 1 were considered as potential biomarkers. After *T*-test, significantly varied (*P* < 0.05) biomarkers were selected as hepatotoxic and nephrotoxic biomarkers, and finally 13 different metabolites were identified by comparison with authentic standards and MS/MS fragments and searching through HMDB, as shown in Table 1.

### 3.5. ROC Curve Analysis

**Figure 5:**
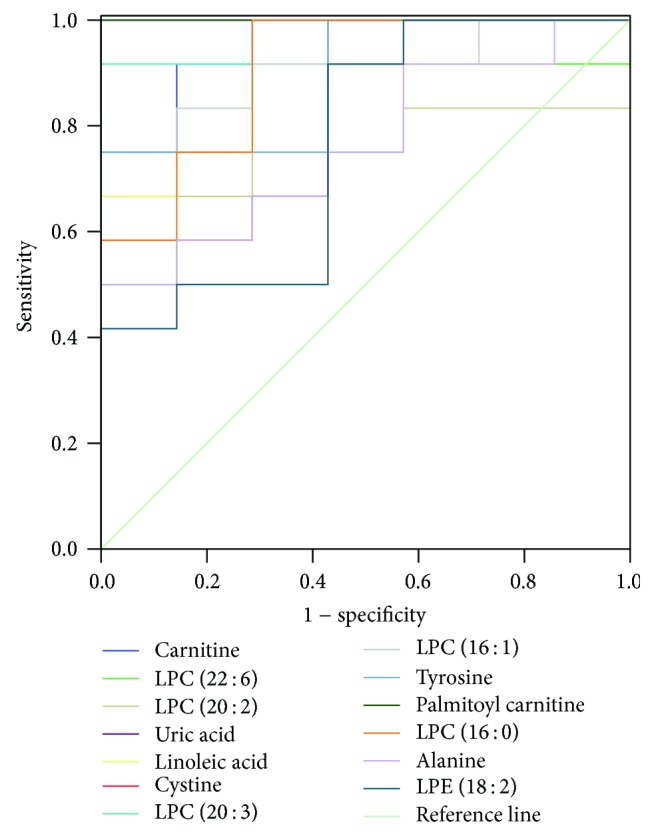
ROC curve of 13 toxic biomarkers.

The ROC curve combines sensitivity and specificity by way of illustration and it is a comprehensive indicator of sensitive and specific continuous variables. The diagnostic efficiency of ROC curve is usually evaluated by the area under the curve (AUC). If AUC is greater than 0.7, the closer the AUC to 1, the better the diagnostic effect. ROC results (Figure 5) showed AUCs of the above-mentioned 13 biomarkers to be all within 0.7–1; thus the 13 biomarkers were determined to be sensitive and specific hepatotoxic and nephrotoxic biomarkers of Psoraleae Fructus. Interestingly, PLD group showed the same change trend of these biomarkers with PMD and PHD groups in comparison to NS group, demonstrating that the untargeted metabonomic method developed in the present study was more sensitive than traditional biochemistry and histopathology.

### 3.6. Metabolomic Pathway Analysis

**Figure 6:**
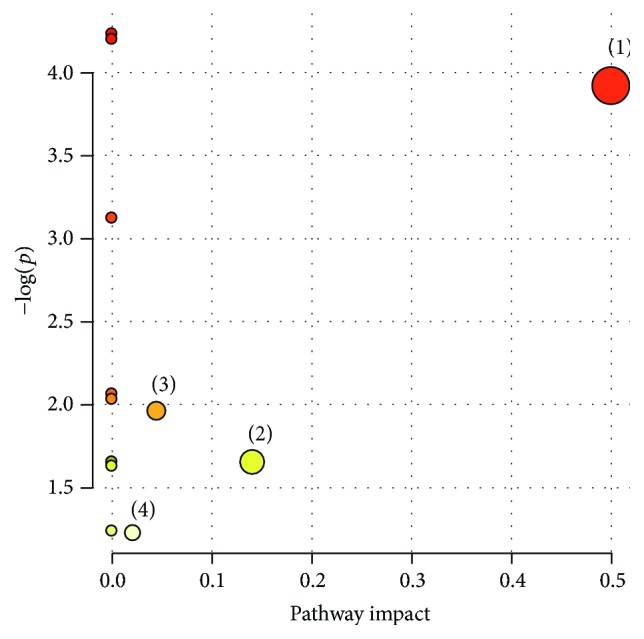
Summary of pathway analysis with MetPA. The interrupted metabolic pathways in Psoraleae Fructus group. (1) Glycerophospholipid metabolism; (2) amino acid metabolism; (3) energy metabolism; (4) purine metabolism.

Metabolomic pathway analysis (MetPA) was performed to find out the metabolic pathways the biomarkers are involved in based on KEGG metabolic pathways. As shown in Figure 6, 13 biomarkers were known to be involved in four metabolic pathways associated with hepatotoxicity and nephrotoxicity of Psoraleae Fructus, which were dysfunctions of glycerophospholipid metabolism, amino acid metabolism, energy metabolism, and purine metabolism.

### 3.7. The Biological Significance of Biomarkers

The occurrence of toxicity of Psoraleae Fructus aqueous extract involved complex* in vivo* metabolic process. Lysophosphatidylcholines (LPCs) are a class of substances from phospholipid, also known as hemolytic lecithin. The synthesis and catabolism of LPCs mainly occur in liver, expressed as the fluctuation of LPCs levels in plasma, so LPCs are of some significance in the diagnosis, prevention, and treatment of liver diseases. PCs are the main components of cell membrane and are produced by LPC catalysis through lecithin cholesterol acyltransferase. When cell membrane undergoes changes, PC content changes to increase the LPC content [15–17].

Meanwhile, it has been reported that plasma LPC levels might be associated with drug-induced nephrotoxicity. Oxidative stress was known to be one of the mechanisms of drug-induced kidney damage [18, 19]. When rats were given nephrotoxic drugs leading to kidney injury, generation of the reactive oxygen species (ROS) and reactive nitrogen species (RNS) was activated, resulting in* in vivo* changes of the oxidative stress levels and out of balance of the body oxidation and antioxidative stress system; consequently varying degrees of nephrocyte damage occurred. PCs can remove excess ROS and RNS in the body, so the body may need to consume a large number of PCs to consume peroxides to achieve normal oxidative stress levels in the case of kidney damage, and the glycerophospholipid metabolism was affected.

Liver acts as the center of amino acid metabolism, which might be disrupted by any liver damage. In our experiment, alanine content was decreased, probably due to the fact that protein synthesis was affected by administration of Psoraleae Fructus to rats [20]. Additionally, as important substances for organic life movement, amino acids are involved in various energy and material metabolism activities. Metabolic dysfunction of amino acids especially branched-chain amino acids will inevitably affect the protein synthesis [21]. In this study, the content of tyrosine was decreased and cystine was increased, which was consistent with the change trends for those reported in nephrotic syndrome [22]. Therefore, it was deduced that content changes of the two amino acids were associated with kidney toxicity caused by Psoraleae Fructus.

Carnitines have been identified as reliable biomarkers to determine abnormality of energy metabolism [23]. Carnitines are relevant to fatty acid metabolism in animals. The main function of carnitines is to transport long chain fatty acids from the outer mitochondrial membrane to the inner membrane as a carrier to promote *β* oxidation of fatty acids and to promote the conversion of lipometabolism into energy [24]. Drug-induced liver damage can lead to abnormal energy metabolism, destroy intracellular mitochondria, increase the *β* oxidation of fatty acid, and elevate the consumption of carnitines, so the plasma palmitoyl carnitine content in the present study decreased.

**Figure 7:**
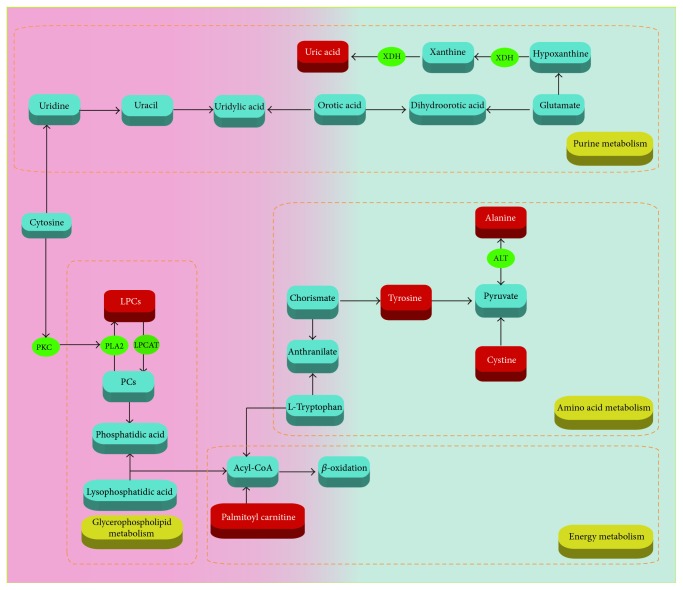
Related metabolic pathways in the Psoraleae Fructus-administrated rats.

Uric acid has a variety of physiological functions in renal injury, including inhibiting nitric oxide bioavailability, reducing renal blood flow, activating renin-angiotensin system, and directly acting on endothelial cells and vascular smooth muscle. Hyperuricemia has been proved to accelerate renal injury and cause irreversible damage to the renal tubules. Increased uric acid levels could induce low density lipoprotein cholesterol oxidation and lipid peroxidation [25]. Uric acid identified as biomarkers in our experiment was changed, indicating that uric acid was related to the renal toxicity of Psoraleae Fructus. Related mechanisms are shown in Figure 7.

## 4. Conclusion

In the present study, toxicity of Psoraleae Fructus in rats was investigated after oral administration of aqueous extract of Psoraleae Fructus at different dosages for consecutive seven days. Untargeted metabonomic method based on UPLC-Q-TOF/MS was applied to evaluate the toxicity of Psoraleae Fructus. The biochemical and histopathological results showed that the medium and high doses of Psoraleae Fructus caused obvious liver and kidney injuries in rats. Metabonomic data were analyzed by multivariate statistical analysis and finally 13 potential biomarkers were identified that might be related to hepatotoxicity and nephrotoxicity induced by Psoraleae Fructus. Through metabolic pathway analysis, it could be concluded that the toxicity of Psoraleae Fructus was attributed to the disruption of glycerophospholipid, amino acid, energy, and purine metabolism. Our results enable people to truly recognize the toxic effects of Psoraleae Fructus and provide scientific guidance for clinical safety assessment of drugs. In addition, the early detection of toxic biomarkers mainly related to glycerophospholipids, amino acid, energy metabolism, and so forth, suggesting that the common mechanism of liver and kidney toxicity may be related to these pathways, and provide theoretical support for the mechanism of toxicity.

## Figures and Tables

**Table 1:** Potential toxic biomarkers of Psoraleae Fructus.

Number	*t*_*R*_ (min)	Obsd *m*/*z*	Calcd *m*/*z*	ppm	Metabolite	Formula	Change trend^a^	MS/MS
(1)	0.80	241.0309	241.0317	−3.32	Cystine	C_6_H_12_N_2_O_4_S_2_	↑	241.0 [M + H]^+^ 121.9 [M + H − C_3_H_5_NO_4_]^+^ 119.9 [M + H − C_3_H_7_NO_4_]^+^

(2)	0.82	162.1134	162.1130	2.47	Carnitine	C_7_H_15_NO_3_	↑	162.1 [M + H]^+^ 103.0 [M + H − C_3_H_9_N]^+^

(3)	0.85	90.0553	90.0549	4.44	Alanine	C_3_H_7_NO_2_	↓	90.0 [M + H]^+^ 72.0 [M + H − H_2_O]^+^

(4)	1.00	169.0360	169.0362	−1.18	Uric acid	C_5_H_4_N_4_O_3_	↓	169.0 [M + H]^+^ 152.0 [M + H − NH_3_]^+^ 126.0 [M + H − CHNO]^+^ 96.0 [M + H − C_2_H_3_NO_2_]^+^

(5)	1.01	182.0819	182.0817	1.10	Tyrosine	C_9_H_11_NO_3_	↓	182.0 [M + H]^+^ 164.1 [M + H − H_2_O]^+^ 136.1 [M + H − C_2_H_6_O]^+^

(6)	5.63	494.3248	494.3247	0.20	LPC (16 : 1)	C_24_H_48_NO_7_P	↑	494.3 [M + H]^+^ 476.3 [M + H − H_2_O]^+^ 184.1 [M + H − C_19_H_34_O_3_]^+^ 104.1 [M + H − C_19_H_35_O_6_P]^+^

(7)	5.80	590.3224	590.3223	0.17	LPC (22 : 6)	C_30_H_50_NO_7_P	↑	590.3 [M + Na]^+^ 568.3 [M + H]^+^ 184.0 [M + H − C_25_H_36_O_3_]^+^ 104.1 [M + H − C_25_H_37_O_6_P]^+^

(8)	5.85	478.2952	478.2934	3.76	LPE (18 : 2)	C_23_H_44_NO_7_P	↑	478.3 [M + H]^+^ 337.3 [M + H − C_2_H_8_NO_4_P]^+^

(9)	6.22	496.3413	496.3403	2.01	LPC (16 : 0)	C_24_H_50_NO_7_P	↑	496.3 [M + H]^+^ 184.0 [M + H − C_19_H_36_O_3_]^+^ 104.1 [M + H − C_19_H_37_O_6_P]^+^

(10)	6.38	546.3564	546.3560	0.73	LPC (20 : 3)	C_28_H_52_NO_7_P	↑	546.4 [M + H]^+^ 528.4 [M + H − H_2_O]^+^ 184.1 [M + H − C_23_H_38_O_3_]^+^ 104.1 [M + H − C_23_H_39_O_6_P]^+^

(11)	6.46	400.3430	400.3427	0.75	Palmitoyl carnitine	C_23_H_45_NO_4_	↓	400.3 [M + H]^+^ 341.2 [M + H − C_3_H_9_N]^+^ 144.1 [M + H − C_16_H_32_O_2_]^+^ 85.0 [M + H − C_19_H_41_NO_2_]^+^

(12)	6.64	303.2322	303.2300	7.26	Linoleic acid	C_18_H_32_O_2_	↑	303.2 [M + Na]^+^ 281.2 [M + H]^+^ 124.0 [M + H − C_9_H_16_O_2_]^+^

(13)	7.12	548.3718	548.3716	0.36	LPC (20 : 2)	C_28_H_54_NO_7_P	↑	548.3 [M + H]^+^ 184.0 [M + H − C_23_H_40_O_3_]^+^ 104.1 [M + H − C_23_H_41_O_6_P]^+^

^a^↑: content increased; ↓: content decreased.
